# Refrigerated modified Knott concentrate enables long-term morphological viability of canine blood microfilariae

**DOI:** 10.29374/2527-2179.bjvm000223

**Published:** 2023-04-25

**Authors:** Leticia Gomes Zanfagnini, Tatiane Paula da Silva, Diefrey Ribeiro Campos, Soraia Figueiredo de Souza, Patrícia Fernandes Nunes da Silva Malavazi, Rômulo Silva de Oliveira, Cíntia Daudt, Acácio Duarte Pacheco

**Affiliations:** 1 Veterinarian, MSc., Programa de Pós-Graduação em Sanidade e Produção Animal Sustentável na Amazônia (PPGESPA), Centro de Ciências Biológicas e da Natureza, Universidade Federal do Acre (UFAC). Rio Branco, AC, Brazil.; 2 Veterinarian, Autonomous, Muriaé, MG, Brazil.; 3 Veterinarian, DSc. Programa de Pós-Graduação em Ciências Veterinárias (PPGCV), Departamento de Parasitologia Animal (DPA). Instituto de Veterinária, Universidade Federal Rural do Rio de Janeiro. Seropédica, RJ. Brazil.; 4 Veterinarian, DSc., Centro de Ciências Biológicas e da Natureza, UFAC. Rio Branco, AC, Brazil.

**Keywords:** diagnosis, *Dirofilaria immitis*, teaching, diagnóstico, *Dirofilaria immitis*, ensino

## Abstract

There are several methods of laboratory diagnosis of filarids, the most used are the thick smear and the Knott method. Both are quick to perform, have a low cost and allow observing the presence, quantifying and analyzing the morphological characteristics of microfilariae. Knowing the morphological viability of fixed microfilariae is of practical importance, as it allows the transport of samples to a laboratory, facilitates epidemiological studies , and allows the storage of samples for didactic. Thus, the aim of this study was to evaluate the morphological viability of microfilariae fixed in the refrigerated modified knott test using 2% formalin. To perform the modified Knott technique, 10 samples of microfilaremic dogs aged over 6 months were used. To evaluate the morphological viability time of the microfilariae in the modified Knott concentrate, the evaluations were repeated after intervals of 0, 1, 7, 30, 60, 120, 180, 240, and 304 days. In the present study, we did not verify any difference in the morphology of the microfilariae in any of the analyzed intervals from day 0 to 304 days, and it is possible to conclude that the use of 2% formalin in the modified Knott technique allows the microfilariae to be identified in a period of 304 days. days after processing the sample without changes in its morphology.

## Resumo

Existem diversos métodos de diagnóstico laboratorial de filarídeos, os mais utilizados são a gota espessa e o método do Knott. Ambos são de rápida execução, possui baixo custo e permitem observar a presença, quantificar e analisar as características morfológicas das microfilárias. Conhecer a viabilidade morfológica das microfilárias fixadas tem importância prática, pois permite o transporte de amostras para um laboratório, facilita estudos epidemiológicos, e permite o armazenamento de amostras para fins didáticos. Desta maneira, o objetivo do trabalho foi avaliar a viabilidade morfológica das microfilárias fixadas em teste do knott modificado refrigerado utilizando formalina a 2%. Para realização da técnica de knott modificado foram utilizadas 10 amostras de cães microfilarêmicos, com idade superior a 6 meses. Para avaliar o tempo de viabilidade morfológica das microfilárias no concentrado de Knott modificado, foram repedidas as avaliações após intervalos de 0,1,7, 30, 60, 120, 180, 240 e 304 dias. No presente estudo não verificamos diferença na morfologia das microfilárias em nenhum dos intervalos analisados deste do dia 0 até 304 dias, sendo possível concluir que a utilização da formalina a 2% na técnica de Knott modificada permite que as microfilárias possam ser identificadas em período de 304 dias após o processamento da amostra sem que ocorram alterações na sua morfologia.

Canine filariasis is a zoonosis with a global distribution and can be caused by several Filaridae, including *Dirofilaria immitis*, *D. repens*, *Acanthocheilonema dracunculoides,* and *A. reconditum* (Bendas et al., 2019; Little et al., 2018; Peribáñez et al., 2001). The identification of filarids with zoonotic potential in non-endemic areas is crucial to public health (De Andrade Vieira et al., 2022; Silva & Langoni, 2009).

Several laboratory methods are available for its diagnosis; the most commonly used are the thick smear and modified Knott's methods. Both are quick and inexpensive to perform, and the modified Knott test is the gold standard, as it allows the visualization, quantification, and analysis of the morphological characteristics of microfilariae, thus facilitating the distinction between different species. (Magnis et al., 2013). Furthermore, the modified Knott technique is the only cost-effective diagnostic test available for *D. repens*, owing to the lack of a commercially available serological test (Moraes et al., 2017).

However, distinguishing between different species can be challenging, requiring a well-trained professional, as they are morphologically similar in mixed infections with different *Dirofilaria* spp. or in cases of low parasitemia. In such cases, molecular or histochemical staining methods are necessary (De Andrade Vieira et al., 2022; Latrofa et al., 2012; Moraes et al., 2017).

Knowing the morphological viability of fixed microfilariae is important as it allows the transport of samples to a specialized laboratory, facilitates epidemiological studies with a large number of samples, provides time for identifying the offending parasites, and allows the storage of samples for didactic purposes because these regions are considered non-endemic, which sometimes hinders the demonstration of microfilariae in practical classes. Thus, this study aimed to evaluate the morphological viability of fixed microfilariae using the modified Knott's test at different times.

Blood samples were previously identified in a research project approved by the Ethics Committee on Animal Use of the Federal University of Acre (CEUA/UFAC), registered under protocol number 05/2021, modified Knott tests were performed to screen for Filaridae in dogsolder than 6 months treated at the Teaching and Research Unit in Veterinary Medicine of the Federal University of Acre.

However, to perform the modified Knott technique, 9.0 mL of 2% formalin was used in a 15-mL Falcon tube, to which 1.0 mL of blood with anticoagulant ethylenediamine tetraacetic acid (EDTA) was added. Subsequently, the material was centrifuged (Coleman® 90-1, Santo André, SP, Brazil) at 2500 rpm for 10 min. The supernatant was discarded, a drop of the sediment (40 µL) was transferred to a glass slide, 10 µL of methylene blue was added, and the slide was covered with a coverslip of 24 × 24 mm. The material was then examined under an optical microscope (Leica ICC50®, Wetzlar, Germany) at 100× and 400× magnification. The presence, number, and morphological characteristics of the microfilariae were evaluated, such as length, tail morphology, and anterior extremity conformation, to help distinguish the species.

Ten samples of microfilaremic dogs were identified using the modified Knott technique and stored to test their morphological viability. Positive Knott concentrates were transferred to 2 mL microtubes without methylene blue and stored under refrigeration at 4°C. Evaluations were repeated at intervals of 1, 7, 30, 60, 120, 180, 240 and 304 days to determine the duration for which the microfilariae would remain morphologically viable in Knott's concentrate.

**Figure 1:**
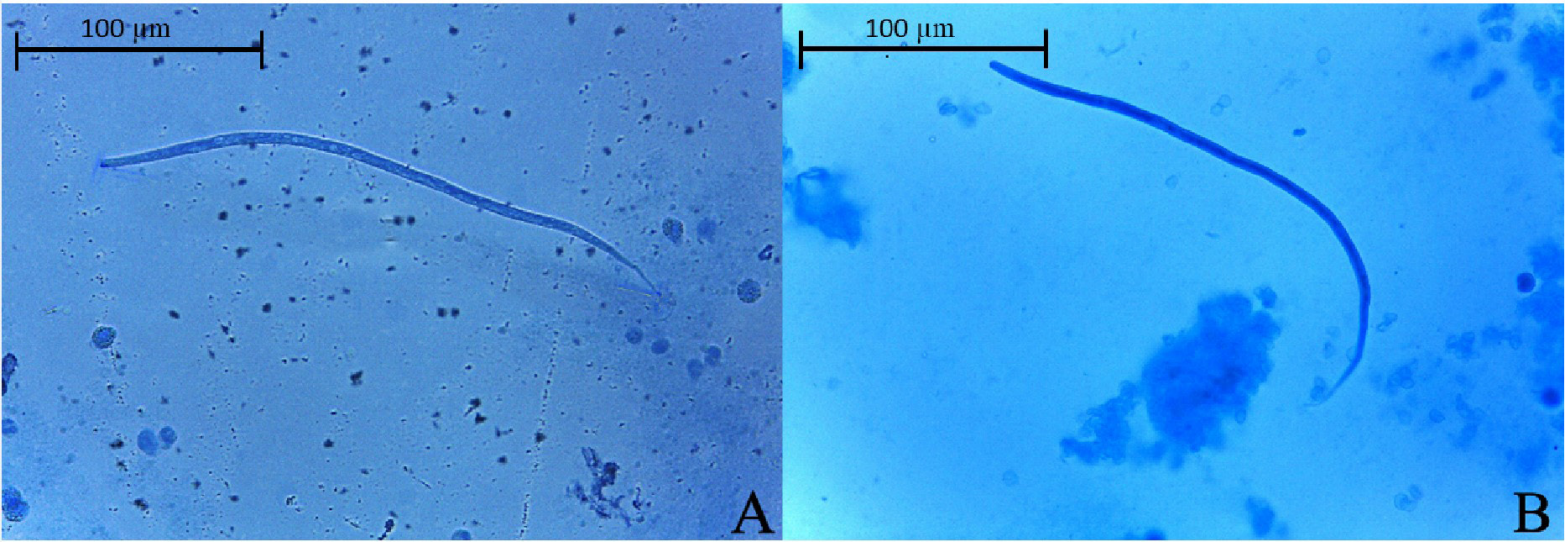
(A) Photomicrograph demonstrating microfilariae on day 0, measuring 242.34 µm; (B) Viable microfilariae from the same knott concentrate as in image (A), after 304 days of conservation, measuring 243.41 µm (Modified Knott, 400X magnification).

No difference was observed in the tail morphology and anterior extremity conformation, there was also no change in microfilariae size (*p*=0.9653) and width (*p*=0.9857) at any of the analyzed intervals from day 0 to 304 (Table 1). However, progressive erythrocyte lysis was observed during the evaluations, whicheased the visualization of microfilariae, as shown in Figure 1.

**Table 1:** Evaluation of the size and width of microfilariae identified by the refrigerated Knott test modified with 2% formalin and on days 0 and 304.

	Day 0		Day 304		p-value
	Average	Standard deviation	Average	Standard deviation	
Length (µm)	247.53	± 16.32	247.85	± 16.06	0.9653
Width (µm)	4.80	± 0.48	4.80	± 0.51	0.9857

Note: The results were submitted to statistical analysis by the unpaired T test (Unpaired T test; Graph Pad Prism 9^®^).

Long et al. (2020) conducted a study to verify the morphological viability of microfilariae by cryopreservation; however, the microfilariae of *D. immitis* obtained a length shorter than that described in the literature and the head score also deacreased as the freezing time increased. Therefore, refrigerating the modified Knott concentrate may have an advantage because it allows the fixation of the microfilariae without dehydrating them and allows future measurements without the size changing. Therefore, the potential for distortions that may contribute to the misidentification of the species is reduced.

In the work carried out by Genchi et al. (2021) despite not mentioning the times evaluated as in our proposal, they used different reagents as safe alternatives to replace 2% formalin in the modified Knott test for the diagnosis of subcutaneous dirofilariasis (*D. repens* ) and cardiopulmonary (*D. immitis* ). Thus, they assessed that the lengths and widths of the microfilariae reduced when they used 2% acetic acid, 2% glacial acetic acid, 10% saponin and distilled water, probably because it caused a more pronounced dehydration of the parasite. When performed with 2% formaldehyde, there was no significant difference (P > 0.05), corroborating the present study. Therefore, methods that do not collaborate with the morphological preservation of filarids will make their classification and, consequently, an assertive diagnosis difficult.

Magnis et al. (2013) evaluated modified Knott concentrates and also found no changes in the microfilarial morphology. However, unlike our study, they reported that the microfilaria remained viable until the 105th day. The authors reported the addition of methylene blue to the concentrate and storage under refrigeration. In our study, methylene blue was added directly to the slides. Therefore, under the conditions of this study, the microfilariae were verifiably visible for a longer time.

In conclusion, refrigerated modified Knott's concentrates using 2% formalin maintain the analytical viability of microfilariae for more than ten months without the deterioration of microfilariae; however, further studies are needed to determine the duration for which they remain viable for analyses.
